# Central Venous Catheter Insertion: A Scoring System for Evaluation of Both the Procedure and the Operator (CVCI Score/Gaber Score)

**DOI:** 10.1155/2020/8156801

**Published:** 2020-11-03

**Authors:** Sayed Gaber, Ahmed Yehia, Beshoy Nabil, Ahmed Samir

**Affiliations:** Critical Care Medicine, Cairo University, Giza, Egypt

## Abstract

**Introduction:**

Currently, there is no method to assess the performance while inserting a central venous catheter. We suggest a new scoring system for evaluation of both the technique as well as the operator, and then we applied it for the comparison between the landmark and ultrasound techniques to assess its validity*. Methods*. Four hundred patients were divided into two equal groups: group (A): internal jugular vein (IJV) and group (B): subclavian vein (SV). The landmark technique and the ultrasound guidance were used equally (100 patients for each) in both groups.

**Results:**

In group (A), 20% of patients in the landmark group achieved score 4, while 82% of patients in the ultrasound group achieved the same score. This suggests that the ultrasound technique for catheterization of IJV decreased overall complications and improved the success rate. In group (B), there were 70% of patients in the landmark group who achieved score 5, while 49% of patients in the ultrasound group achieved the same score which proposes that the landmark technique might be deceptively better than the ultrasound technique for catheterization of SV. This could be because the time required for catheterization of SV by the ultrasound technique was longer than that in the landmark technique. Overall complications of 15% with the landmark technique vs. 2% with ultrasound guidance in this group of patients are not only statistically significant but also increase morbidity and mortality with a highly invasive procedure. Complications and their incidences are by far more significant than seconds of time. Our results suggest that the ultrasound technique could decrease the incidence of overall complications, but it is time-consuming in group (B). These results support the validity of our new scoring system.

**Conclusion:**

We suggest a new scoring system for CVC insertion that can be used for evaluation of both the technique and the operator. It can evaluate the performance of junior staff and follow their progress. It can be applied in the medical and critical care practice as well as the quality management privileges and protocols.

## 1. Introduction

Currently, there is no method to assess the performance while inserting a central venous catheter (CVC). We tried to initiate a new scoring system that has four limbs. It can evaluate the technique as well as the operator. Then, we tried to apply it for the comparison between the landmark and ultrasound techniques to assess its validity as there is now growing interest in the use of the ultrasound guidance for placement of CVCs in ICU patients [1].

### 1.1. Patients and Methods

Four hundred consecutive patients who were admitted to the Critical Care Department, Kasr Al-Aini, from January 2015 to October 2016, were included in this prospective study. They have been randomly divided into two equal groups:

  Group (A): two hundred patients, in whom catheterization of internal jugular vein (IJV) was done using two different techniques
  Group (A1): one hundred patients; CVCs were inserted into the IJV using the landmark technique
  Group (A2): one hundred patients; CVCs were inserted into the IJV using ultrasound guidance
  Group (B): two hundred patients, in whom catheterization of subclavian vein (SV) was done using two different techniques
  Group (B1): one hundred patients; CVCs were inserted into the SV using the landmark technique
  Group (B2): one hundred patients; CVCs were inserted into the SV using ultrasound guidance

### 1.2. Inclusion Criteria

Adult patients >18 years old were admitted to the critical care unit and required central venous catheter insertion.Informed consent was written by the patient or his\her next of kin.

### 1.3. Exclusion Criteria

- Patients during cardiopulmonary resuscitation
- Patients having central venous catheter in place
- Patients with significant coagulopathy (INR > 2, platelet count < 30.000, and PTT > 60 seconds)

### 1.4. Equipment

1. Catheters: in the present study, two lumen catheters were used
2. Ultrasound devices: in the present study, Vivid e GE Ultrasound was used with high-frequency linear probe, 7.5 MHz

## 2. Methods

(1) Consent: after approval of the local ethics committee, written informed consent was obtained from each patient or relatives.
(2) Patient data:
    Demographic data: age, sex, weight, height, and BMI were recorded (obesity was defined as BMI more than 30).
    History of previous catheterization.
    Investigations include the following:
    (a) Complete blood count, (b) coagulation profile including P.T., PTT, INR, bleeding time, and coagulation time, and (c) plain chest X-ray.

### 2.1. Statistical Analysis of the Data

Data were fed into the computer and analyzed using IBM SPSS software package version 20.0. Qualitative data were described using number and percent. Quantitative data were described using range (minimum and maximum), mean, standard deviation, and median. Significance of the obtained results was judged at the 5% level.

The used tests were

1. Chi-square test:
2. For categorical variables to compare between different groups
3. Fisher's exact or Monte Carlo correction:
4. For chi-square when more than 20% of the cells have expected count less than 5
5. Student's *t*-test:
6. For normally quantitative variables to compare between two studied groups
7. Mann–Whitney test:
8. For abnormally quantitative variables to compare between two studied groups

## 3. Results

### 3.1. Group (A): IJV Catheterization

Landmark group (A1) included 100 patients with a mean age of 65.13 ± 12.80 years old (ranged from 38 to 92 years). Sixty-nine patients (69%) were males, and 31 patients (31%) were females.

However, in ultrasound group (A2), the number was 100 patients with a mean age of 62.23 ± 11.19 years old (ranged from 36 to 87 years). Sixty-eight patients (68%) were males, and 32 patients (32%) were females.

There were no significant differences between the landmark group and the ultrasound group according to age, sex, and BMI.

The results in Table 1 showed that ultrasound-guided technique for catheterization of IJV could decrease the number of attempts, reduce the time of cannulation, decrease the incidence of complications, and improve the success rate.

Table 2 shows significant difference between the two subgroups in group (A) as regards the number of attempts and time of cannulation.

Table 3 shows significant difference between the two subgroups in group (A) as regards the number of overall complications.

Table 4 shows that the success rate was significantly increased when using the ultrasound guidance technique in comparison to the landmark technique in the IJV group (*p*=0.035).

### 3.2. Group (B): SV Catheterization

Landmark group (B1) included 100 patients with a mean age of 59.31 ± 13.99 years old (ranged from 35 to 91 years). Sixty-eight patients (68%) were males, and 32 patients (32%) were females. However, in ultrasound group (B2), the number of patients was 100 with a mean age of 61.02 ± 12.64 years old (ranged from 29 to 89 years). Sixty-four patients (64%) were males, and the remaining 36 patients (36%) were females. There were no significant differences between the landmark group and the ultrasound group according to age, weight, and BMI.

The results in Table 5 showed that there were 70% of patients in the landmark group who achieved score 5; this was in contrast to 49% of patients in the ultrasound group who achieved the same score (*p*=0.002), which suggests that the landmark technique might be better than the ultrasound technique, and this may be because the time required for catheterization of SV by the ultrasound technique was much longer than that in the landmark technique.

Table 6 shows significant difference between the two subgroups in group (B) as regards the time of cannulation.

Table 7 shows that there was significant reduction in overall complications between the landmark technique group (15/100) and the ultrasound guidance group (2/100). *p*=0.001 in group (B).

Table 8 shows that there was no statistical significant difference in success rate between the landmark group and the ultrasound group (*p*=0.194). The success rate was 93% in the landmark group, while in the ultrasound group, it was 97%.

## 4. Discussion

### 4.1. Central Venous Catheter Insertion Score (CVCI Score or Gaber Score)

We tried to initiate a new scoring system for CVC insertion depending on the data in our study.

  The suggested scoring system has 4 limbs: 1, number of attempts, 2, time of cannulation, 3, complications, and 4, success or failure.
  Regarding the number of attempts: if the CVC was inserted after one attempt, this was given one point, after 2 attempts, 2 points, and after more than 2 attempts, 3 points.
  Regarding the time of cannulation: the cannulation time in our study is defined as the time lapse from the start of skin puncture to aspiration of blood from the catheter in seconds. If the CVC was inserted in 90 seconds or less, this was given one point, from 91 seconds to 180 seconds, 2 points, and more than 180 seconds, 3 points.
  Regarding complications: if the CVC was inserted without any complications, this was given one point, one complication occurred, 2 points, and more than one complication occurred, 3 points.
  Regarding success or failure: if the CVC was inserted successfully, this was given one point, and if the CVC was failed to be inserted, this was given 2 points.

The patients enrolled in our study were classified according to the 4 limbs of our scoring system.

  The best score was 4, which means that the CVC was inserted successfully from the first attempt in 90 seconds or less without any complications.
  The worst score was 11, which means that the CVC was failed to be inserted after more than 2 attempts, time required for insertion was more than 180 seconds, and finally, the patient had more than one complication.
  Group (A): the internal jugular vein group: only 20% of patients in the landmark group achieved the best score (score 4), which means that CVCs were inserted successfully from the first attempt in 90 seconds or less without any complications. In contrast to this, 82% of patients in the ultrasound group achieved the same score (*p* < 0.001) < as shown in Table 1.
  These results showed that the ultrasound-guided technique for catheterization of IJV could decrease the number of attempts, reduce the time of cannulation, decrease the incidence of complications, and improve the success rate with significant difference between the two subgroups as shown in Tables 2–4.
  Group (B): the subclavian vein group: there were 70% of patients in the landmark group who achieved score 5; in contrast to this, 49% of patients in the ultrasound group achieved the same score (*p*=0.002), as shown in Table 5, which proposes that the landmark technique might be deceptively better than the ultrasound technique for catheterization of SV. This could be because the time required for catheterization of SV by the ultrasound technique was much longer than that in the landmark technique with significant difference between the two subgroups as regards the time of cannulation as seen in Table 6.
  These results suggest that the ultrasound-guided technique for catheterization of SV could decrease the overall complication rate in comparison to the landmark technique as seen in Tables 7 and 8. However, the ultrasound-guided technique had increased the time for catheterization of SV than the landmark technique.

### 4.2. Group (A): The Internal Jugular Vein Groups

In this study, in the internal jugular vein group, cannulation was successful in 92% in the landmark group and 99% in the ultrasound group with a statistically significant *p* value (*p*=0.035). In the present study, there were 8 cases of failure in the landmark group; however, in the ultrasound group, there was one case of failure due to preexisting thrombus formation discovered by ultrasound guidance that could be considered as success, not failure. The success rate in the landmark group is lower than that reported by Karakitsos et al. [1] (94.4%); this could be because their patients were mechanically ventilated and sedated so that irritability and unwanted movements by the patients were absent, and if the first attempt of catheter insertion failed, another physician performed the next attempt.

The cannulation time of the patients in the landmark group, in our study, ranged between 90 and 420 seconds with a mean of 125.6 ± 72.74 seconds, while in the ultrasound-guided group, the cannulation time ranged between 60 and 180 seconds with a mean of 80.87 ± 22.73 seconds. This time was significantly shorter in the ultrasound-guided group than that in the landmark group (*p* < 0.001) (Table 2).

Both the present study and the previous studies were similar in that cannulation time was significantly shorter in the ultrasound group than that in the landmark group, but there were some points of differences such as the cannulation time in Karakitsos et al.'s study [1] was shorter than that in our study because there was difference in the definition of cannulation time (access time) in both studies. In our study, the cannulation time is defined as time lapse from the start of skin puncture to aspiration of blood from the catheter in seconds, but in Karakitsos et al.'s study [1], access time was defined as the time between penetration of skin and aspiration of venous blood into the syringe. So, the mean time of cannulation in Karakitsos et al.'s study [1] was shorter than that in the present study.

In our study, the number of attempts in the landmark group ranged between 1 and 6 with a mean of 1.54 ± 1.03, while in the ultrasound group, the number of attempts ranged between 1 and 3 with a mean of 1.21 ± 0.50. There was a significant reduction in the number of attempts between landmark and ultrasound groups (*p*=0.011) (Table 2), which was comparable to the study done by Agarwal et al. [2] and also in many previous studies such as Parajuli and Pokharel [3] and Karakitsos et al. [1].

These results indicated that the ultrasound guidance is better than the landmark technique in IJV central venous line insertion.

### 4.3. Group (B): The Subclavian Vein Groups

In this study, the subclavian vein cannulation was successful in 93% in the landmark group and 97% in the ultrasound group (*p*=0.194) (Table 8). There was no statistical significant difference in success rate between the landmark and ultrasound groups. This is similar to the studies done by Alice et al. [4], Palepu et al. [5], and Oh et al. [6]. The success rate in the landmark group in our study was higher than that reported by Fragou et al. [7]. This could be due to the large number of patients, about 200 patients, in each group in Fragou et al.'s study [7].

In the ultrasound group, our results were similar to that reported by Oh et al. [6], but the success rate was lower than that reported by Fragou et al. [7] (100%). This could be due to patients in Fragou et al. [7] study were mechanically ventilated and the procedure was done under general anesthesia.

Cannulation time of the patients in the landmark group in our study ranged between 110 and 450 seconds with a mean of 150.39 ± 73.28 seconds and median 118, while in the ultrasound-guided group, the cannulation time ranged between 150 and 400 seconds with a mean of 200.49 ± 68.45 seconds and median 180 (*p* < 0.001) (Table 6). The cannulation time is significantly longer in the ultrasound group than that in the landmark group which was comparable to Alice et al.'s study [4].

In the landmark group, the overall complication rate in our study was 15% (Table 7). The result was similar to Palepu et al.'s study [5], but it was lower than the rate reported by Fragou et al. [7]. This may be because many complications were included in Fragou et al.'s study [7] more than our study such as catheter misplacement, injury of the brachial plexus, phrenic nerve injury, and cardiac tamponade.

In the ultrasound group, the overall complication rate in this study was 2% (Table 7). The result was lower than that reported by Palepu et al. [5], Alice et al. [4], and Fragou et al. [7]. However, by excluding the other complications of Fragou et al.'s study [7] that were not done in our study, the rate of overall complications was similar (2%).

These results showed that, in comparison with the landmark technique, despite the time of catheterization of SV was significantly longer with ultrasound guidance than with the landmark technique, the overall complications were significantly decreased when using the ultrasound guidance. Overall complications of 15% with the landmark technique vs. 2% with ultrasound guidance in this group of patients are not only statistically significant but increase morbidity and mortality with a highly invasive procedure. Complications and their incidences are by far more significant than seconds of time.

These results support the validity of our new scoring system that can be applied in the medical and critical care practice as well as the quality management privileges and protocols.

## 5. Conclusion

We suggest a new scoring system for CVC insertion (Gaber score) that can be used for evaluation of both the technique as well as the operator. It can evaluate the performance of junior staff and follow their progress. It can be applied in the medical and critical care practice as well as the quality management privileges and protocols.

## Figures and Tables

**Table 1:** Group (A): comparison between landmark and ultrasound (IJV) techniques according to our scoring system.

Score	Landmark IJV (*n* = 100)		Ultrasound IJV (*n* = 100)		*χ* ^2^	*p*
	No.	%	No.	%		
4	20	20.0	82	82.0	76.911^*∗*^	<0.001^*∗*^
5	42	42.0	2	2.0	46.620^*∗*^	<0.001^*∗*^
6	16	16.0	8	8.0	3.030^*∗*^	0.082
7	9	9.0	5	5.0	1.229	0.268
8	4	4.0	3	3.0	0.148	^FE^ *p*=1.000
9	2	2.0	0	0.0	2.020	^FE^ *p*=0.497
10	6	6.0	0	0.0	6.186^*∗*^	^FE^ *p*=0.029^*∗*^
11	1	1.0	0	0.0	1.005	^FE^ *p*=1.000

^∗^: Statistically significant at *p* ≤ 0.05. FE: Fisher Exact for Chi square test.

**Table 2:** Comparison between the landmark and ultrasound techniques in IJV group (A) according to the number of attempts and time of cannulation.

	Landmark IJV (*n* = 100)	Ultrasound IJV (*n* = 100)	*Z*	*p*
*Number of attempts*				
Min–Max	1.0–6.0	1.0–3.0	2.553^*∗*^	0.011^*∗*^
Mean ± SD	1.54 ± 1.03	1.21 ± 0.50		
Median	1.0	1.0		
*Time of cannulation*				
Min–Max	90.0–420.0	60.0–180.0	8.965^*∗*^	<0.001^*∗*^
Mean ± SD	125.65 ± 72.74	80.87 ± 22.73		
Median	98.0	73.50		

FE: Fisher Exact for Chi square test. ^∗^: Statistically significant at *p* ≤ 0.05.

**Table 3:** Comparison between landmark and ultrasound techniques in IJV group (A) according to complications.

Complication	Landmark IJV (*n* = 100)		Ultrasound IJV (*n* = 100)		*χ* ^2^	*p*
	No.	%	No.	%		
Arterial punctures	16	16.0	4	4.0	8.000^*∗*^	0.005^*∗*^
Hematoma	12	12.0	2	2.0	7.680^*∗*^	0.006^*∗*^
Hemothorax	0	0.0	0	0.0	–	–
Pneumothorax	2	2.0	0	0.0	2.020	^FE^ *p*=0.497
Overall complications	27	27.0	6	6.0	16.004^*∗*^	<0.001^*∗*^

FE: Fisher Exact for Chi square test. ^∗^: Statistically significant at *p* ≤ 0.05.

**Table 4:** Comparison between landmark and ultrasound techniques in IJV group (A) according to success rate.

Success rate	Landmark IJV (*n* = 100)		Ultrasound IJV (*n* = 100)		*χ* ^2^	*p*
	No.	%	No.	%		
Success rate	92	92.0	99	99.0	5.701^*∗*^	^FE^ *p*=0.035^*∗*^
Failure rate	8	8.0	1	1.0	5.701^*∗*^	^FE^ *p*=0.035^*∗*^

FE: Fisher Exact for Chi square test. ^∗^: Statistically significant at *p* ≤ 0.05.

**Table 5:** Group B: comparison between landmark and ultrasound (SV) techniques according to our scoring system.

Score	Landmark SV (*n* = 100)		Ultrasound SV (*n* = 100)		*χ* ^2^	*p*
	No.	%	No.	%		
4	0	0.0	0	0.0	—	—
5	70	70.0	49	49.0	9.150^*∗*^	0.002^*∗*^
6	9	9.0	30	30.0	14.047^*∗*^	<0.001^*∗*^
7	5	5.0	15	15.0	14.047^*∗*^	0.018^*∗*^
8	7	7.0	2	2.0	2.909	^FE^ *p*=0.170
9	5	5.0	3	3.0	0.521	^FE^ *p*=0.721
10	2	2.0	1	1.0	0.338	^FE^ *p*=1.000
11	2	2.0	0	0.0	2.020	^FE^ *p*=0.497

FE: Fisher Exact for Chi square test. ^∗^: Statistically significant at *p* ≤ 0.05.

**Table 6:** Comparison between landmark and ultrasound in SV group (B) according to the number of attempts and time of cannulation.

	Landmark SV (*n* = 100)	Ultrasound SV (*n* = 100)	*Z*	*p*
*Number of attempts*				
Min–Max	1.0–6.0	1.0–3.0	0.871	0.384
Mean ± SD	1.46 ± 0.99	1.27 ± 0.55		
Median	1.0	1.0		
*Time of cannulation*				
Min–Max	110.0–450.0	150.0–400.0	7.783^*∗*^	<0.001^*∗*^
Mean ± SD	150.39 ± 73.28	200.49 ± 68.35		
Median	118.0	180.50		

FE: Fisher Exact for Chi square test. ^∗^: Statistically significant at *p* ≤ 0.05.

**Table 7:** Comparison between landmark and ultrasound in SV group (B) according to complications.

Complications		Landmark SV (*n* = 100)		Ultrasound SV (*n* = 100)		*χ* ^2^	^FE^ *p*
		No.	%	No.	%		
Arterial puncture		6	6.0	1	1.0	3.701	0.118
Hematoma		5	5.0	1	1.0	2.749	0.212
Hemothorax		0	0.0	0	0.0	–	–
Pneumothorax		6	6.0	1	1.0	3.701	0.118
Overall complications		15	15.0	2	2.0	10.865^*∗*^	^*χ*^2^^ *p*=0.001^*∗*^

FE: Fisher Exact for Chi square test. ^∗^: Statistically significant at *p* ≤ 0.05.

**Table 8:** Comparison between landmark and ultrasound in SV group (B) according to success rate.

Success rate	Landmark SV (*n* = 100)		Ultrasound SV (*n* = 100)		*χ* ^2^	*p*
	No.	%	No.	%		
Success rate	93	93.0	97	97.0	1.684	0.194
Failure rate	7	7.0	3	3.0	1.684	0.194

## Data Availability

The table data used to support the findings of this study are available from the corresponding author upon request.
